# Clinical Significance of Cancer Stem Cell Markers *CD133* and *CXCR4* in Osteosarcomas

**DOI:** 10.31557/APJCP.2020.21.1.67

**Published:** 2020

**Authors:** Azam Mardani, Elmira Gheytanchi, Seyed Hamzeh Mousavie, Zahra Madjd Jabari, Tina Shooshtarizadeh

**Affiliations:** 1 *Department of Pathology, *; 2 *Oncopathology Research Center, *; 3 *Department of Surgery, Rasool-Akram Hospital, Iran University of Medical Science, Tehran, Iran. *

**Keywords:** Osteosarcoma, CD133, CXCR4, bone cancer, prognosis

## Abstract

**Objective:**

Osteosarcomas (OS) is one the most common primary bone malignancy in humans with the lungs metastasis in most cases. Metastasis and recurrence of OS is attributed to cancer stem cells (CSCs). Our study aimed to evaluate the clinical significance of *CD133* and C-X-C chemokine receptor type 4 (*CXCR4*) as the frequently applied markers for CSCs in OS patients.

**Methods:**

In this cross-sectional, a total of 50 tissue samples from the patients with primary OS were immunohistochemically examined to detect the expression of *CD133* and *CXCR4*. The associations of the relative expression and clinical significance of each marker were also evaluated.

**Results:**

High level expression of *CD133* was detected in 26% of OS patient tissues. Of the 12 patients who showed lung metastasis, 5 cases showed high expression of *CD133* with marginal trend correlation (P=0.06). No significant correlation was observed between *CD133* expression and clinicopathological factors. Only 36% of cases showed *CXCR4* expression which was not significantly correlated with gender, age, tumor size, necrosis, stage and metastasis (P>0.05). Clinically, patients with concomitant *CD133/CXCR4 *expression had significant association with lung metastasis (P=0.05).

**Conclusion:**

Our findings showed that concomitant expression of CSC markers *CD133/CXCR4* might had a synergistic effect on the OS poor prognosis. These markers could be considered as potential therapeutic candidates of OS targeted therapy.

## Introduction

Bone cancers are a group of mesenchymal malignancies that include osteosarcoma (OS) and chondrosarcoma with clinical, histological, and molecular heterogeneity (Gibbs et al., 2005). OS, also called osteogenic sarcoma, is the most common malignant primary bone tumor in humans which is exhibited with the lungs micro metastasis as the main causes of OS deaths at diagnosis in approximately 95% of the patients (Adhikari et al., 2010). The most common sites for OS are the distal femoral, proximal tibia and proximal humeral metaphysis (Lu et al., 2015c). The age-standardized incidence of osteosarcoma is estimated at approximately 5 per million annually which is typically occurs in children, adolescents and young adults (He et al., 2012; Ottaviani and Jaffe, 2009). The lung considered as the most common distant metastatic site for OS with a high-grade histopathology.

Potential for spontaneous pulmonary metastasis has been reported in the established osteosarcoma cell lines such as 9607-F5M2 (F5M2) with high tumorigenic ability. Despite multidisciplinary therapeutic strategies including intensive or neoadjuvant chemotherapy specially for high-grade osteosarcoma after aggressive surgical resection (mostly limb-sparing) and radiotherapy, the survival rate of osteosarcoma patients is still low (Adhikari et al., 2010). Therefore, in order to design appropriate therapeutic strategies and to overcome OS tumor progression, it is essential to understand the molecular mechanisms underlying chemo resistance and metastasis (Tobe et al., 2017).

It has been suggested that OS cancer stem cells (CSCs) may play roles in tumor recurrence, metastasis and chemo resistance (Yan et al., 2016). CSCs are a subpopulation of tumor cells with self-renewal and differentiation properties that can sustain tumor growth and recapitulate a heterogeneous tumor (Keymoosi et al., 2014; Madjd et al., 2013). Treatment failure and recurrence in several cancers including OS are attributed to the lack of ability in CSCs targeting (Adhikari et al., 2010). Therefore finding novel diagnostic and prognostic biomarkers to target these cells result in the successful treatment of OS (Yan et al., 2016). The functional methods of CSC detection in different types of sarcomas based on cell surface markers and also in stem-like gene expression levels have been studied in previous studies (Gibbs et al., 2005; Diestra et al., 2002; Murase et al., 2009; Suvà et al., 2009; Adhikari et al., 2010; Terry and Nielsen, 2010;Sana et al., 2011). 

Different stem-like genes and markers including *ALDH*, *CD133*, *CD117/Stro1*, *CD271*, *Oct-4*, *NANOG*, *CXCR4*, nestin and *ABCG2* related with the CSC phenotype have been reported by previous studies (Brown et al., 2017; Honoki et al., 2010; Wang et al., 2011; Tirino et al., 2008; Tirino et al., 2011; Adhikari et al., 2010). Adhikari et al evaluated mesenchymal stem cell (MSC) markers such as* CD117* and Stro-1in OS CSCs duo to its mesenchymal origin and found that theses markers could be easily initiated tumors in immunocompromised mice (Adhikari et al., 2010). They showed that all of *CD117+ *highly metastatic cells with drug-resistant properties were positive for *CXCR4* and *ABCG2* (Adhikari et al., 2010). One of CSC markers which involved in hematopoietic stem cell (HSC) mobilization (Gelmini et al., 2008) and homing and other cancers metastasis (Zhang et al., 2012; Hermann et al., 2007) is the chemokine SDF1/*CXCL12* and its receptor *CXCR4* (Adhikari et al., 2010). The stromal cell-derived factor-1 (*SDF-1/CXCR4*) axis has been considered as a seven-transmembrane G protein-coupled receptor which led to tumor metastasis in several cancers (Kucia et al., 2005; Müller et al., 2001; Saur et al., 2005; Zeelenberg et al., 2003; Lu et al., 2015b). Therefore, *CXCR4* expression has been linked to tumor cell invasiveness (Wang et al., 2015; Song et al., 2015; Perissinotto et al., 2005; Sun et al., 2010) which is also expressed by osteoblasts and by malignant cells in osteosarcoma (Jung et al., 2006).

Therefore, cumulative evidence proposes a critical role of *CXCR4* as a CSC marker in the metastasis. It has been shown that *CXCR4* was expressed and enriched in *CD133 *glioblastoma CSCs (Liu et al., 2006). *CD133* (also known as prominin-1/ AC133), is a member of the Penta span transmembrane glycoproteins and a cell surface marker with a molecular weight of 120 kDa (Madjd et al., 2016; Madjd et al., 2013). It is firstly expressed in hematopoietic stem cells (He et al., 2012) and is also considered as the most important surface marker for identification of CSCs in various solid tumors, such as hepatocarcinoma (Yin et al., 2007), colorectal cancer (Kazama et al., 2017), lung cancer (Roudi et al., 2015), transitional cell carcinomas (Sedaghat et al., 2017), synovial sarcoma and melanoma (He et al., 2012; Madjd et al., 2013). He et al showed that high expression of *CD133* in OS tissues could predicts lung metastasis, poor prognosis and short survival time in OS patients which were correlated with higher expression of other well-known CSC markers including Oct-4, NANOG and *CXCR4* in gene expression levels (He et al., 2012). Current study is focused on the immunohistochemically analysis of CXCR4 and CD133 expression in osteosarcoma as the most frequent types of adult and pediatric sarcomas. Up to now, this is the first report concerning combine analysis of these two putative CSC markers (CXCR4/CD133) in osteosarcomas. Moreover, we also evaluated the possible relationship of *CXCR4* and *CD133* expression in osteosarcoma samples. 

## Materials and Methods

*Patient’s data collection and tumor characteristics*

In this cross-sectional, formalin- fixed-paraffin-embedded (FFPE) OS sections from 50 patients who were diagnosed with primary OS and who underwent tumor resection between 2011–2016 years were obtained from the Shafa-Yahyaian Hospital , a major university-based and referral center in Tehran, Iran.

All patients had written informed consent for participation in the present study. Furthermore, the ethical use of patients’ tissue blocks and the chart reviews were approved by the Ethics Committee of the Iran University of Medical Sciences.

Postoperative adjuvant chemotherapy [four/six courses of Ifosfamide (2 g/m^2^ for 5 days/course), methotrexate (8 g/m^2^ for 1 day/course) and Adriamycin (50 mg/m^2 ^for 1 day/course)] were only prescribed in patients with Enneking stage I, II, III or IV disease. Patient’s medical records were reviewed to collect relevant clinical data, including gender, age, tumor location, tumor size, Enneking stage, necrosis, local recurrence and lung metastasis status. All hematoxylin and eosin stained tumor slides were evaluated for diagnosis by experienced pathologist.

*Immunohistochemistry (IHC) assay*

The level of *CD133* and *CXCR4* expression was examined in OS samples by IHC technique, as described previously (Foroozan et al., 2017; Sedaghat et al., 2017). Briefly, paraffin-embedded tissues (5-μm thickness) were mounted onto Super frost slides (Superfrost plus, Thermo Scientific, Germany), dewaxed at 60^o^C for 30 min, deparaffinized in xylene and rehydrated in different concentrations of ethanol. The sections then were treated by 3% hydrogen peroxide for 20 min to quench the endogenous peroxidase activity. Antigenic retrieval process was performed by submerging in Tris-EDTA (pH 8.0) citrate buffer (pH 6.0) as a target retrieval solution of *CXCR4* and *CD133*, respectively to damask antigenic epitope. The slides were then allowed to cool at room temperature, followed by rinsing in Tris-buffered saline (TBS) and then in protein block serum-free (Dako; code X0909; Denmark; Ready-to-use) to decrease nonspecific binding and background staining for 10 min. For primary staining, all slides were incubated in monoclonal mouse anti-human CD133 antibody (1:100, overnight at 4^o^C, Sina Biotech; SB-019762; Iran) and monoclonal mouse anti-human CXCR4/CD184 (ready to-use, 60 min at RT, Medaysis; code RM0407RTU7), respectively. For secondary staining, EnVisionTM/HRP, Rabbit/Mouse (ENV) reagent (Dako; code K5007; Denmark; Ready to-use) and then visualized by Dako REALTM DAB+ Chromogen (Dako; Denmark) according to the manufacturer’s instructions. After washing, the sections were followed by counterstaining with Mayer’s hematoxylin dye (Dako; Denmark) for 15 min, dehydrated in different degrees of alcohol and cleared in xylene. Human tonsil and kidney tissues were used as positive controls of *CXCR4* and *CD133* antibodies, respectively. For negative control staining the primary antibodies were omitted. 

*Evaluation and scoring system of immunohistochemistry*

Each stained sections were evaluated by two independent pathologist observers (T.S. and A.M.) without knowledge of the patient’s outcome and other clinical characteristics and scored based on the intensity and extensity of staining, and histochemical score (H-score) by multiplying the staining intensity and extensity of positive tumor cells. For each marker, membranous and cytoplasmic immunostaining was considered to be positive. The scoring of CD133 staining (0-300) was classified as the staining extension (0-100) multiplied by the staining intensity (0-3). The extensity of *CXCR4+ *tumor cells (TC) was categorized into four levels: 1 (25 % *CXCR4+* TC), 2 (26–50 % *CXCR4+ *TC), 3 (51–75 % *CXCR4+ *TC), and 4 (76–100 %* CXCR4+* TC). The intensity of immunostaining was classified as no staining (0/-), weak or only visible at high magnification 40× (1+), moderate or visible at low magnification 10× (++), and strong at low magnification 10× (+++). The whole sections were screened under light microscope at ×400 magnification and were classified into Low or high expression levels of *CXCR4* and *CD133* based on the median of H-scores as a cut-off value (Keymoosi et al., 2014). 

*Statistical analyses*

Each experiment was analyzed using the SPSS software version 20 (SPSS, Chicago, IL, USA). Values were stated as mean ± SD. Correlation of each marker with clinicopathological features of patients was evaluated using Pearson’s chi-square or Fisher’s exact test, where appropriate. The one-way ANOVA and Tukey post hoc analysis was applied to examine the association between CD113/CXCR4 phenotypes and clinicopathological variables. A value of P < 0.05 was considered statistically significant. 

## Results

*Patient clinicopathological characteristics*

The mean age of all OS cases was 19±8.926 years ranging from 10-60 and 24 (48%) patients were older than 19 years of age. Twenty-nine (58%) of patients were male and 21 (42%) of them were female. OS tumors were located in right/left dis femur, right/left tibia, right foot and shoulder, right/left proximal fibula, right/left humerus, and left pelvic locations. In total, 23 (46%) patients had less than 90% and 27 (54%) of them had more than 90% necrosis. Of the 50 patients, 38 (76%) cases had no metastases and 10 (20%) cases had lung metastases and iliac and lumbar spine metastases was only observed in 1 and 1 (2%) of cases. Regarding Enneking staging, 38 (76%) patients were at stage II and 12 (24%) at stage III. 

*Correlation between CD133 expression and clinicopatho¬logical characteristics*

**Figure 1:**
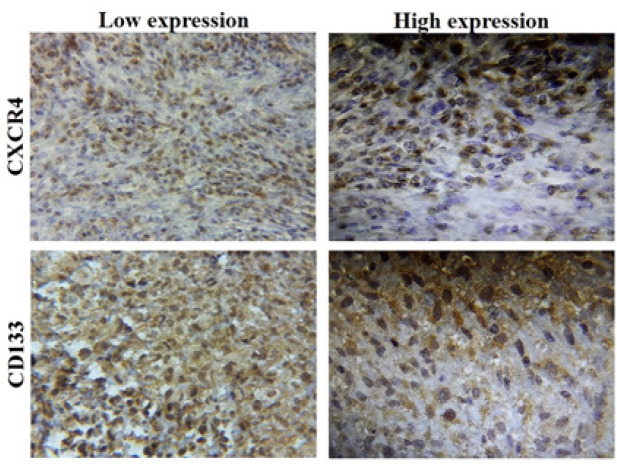
Low and High CD133 and CXCR4 Expression Patterns in Human OS Tissue (Magniﬁcation, 400 ×)

**Figure 2:**
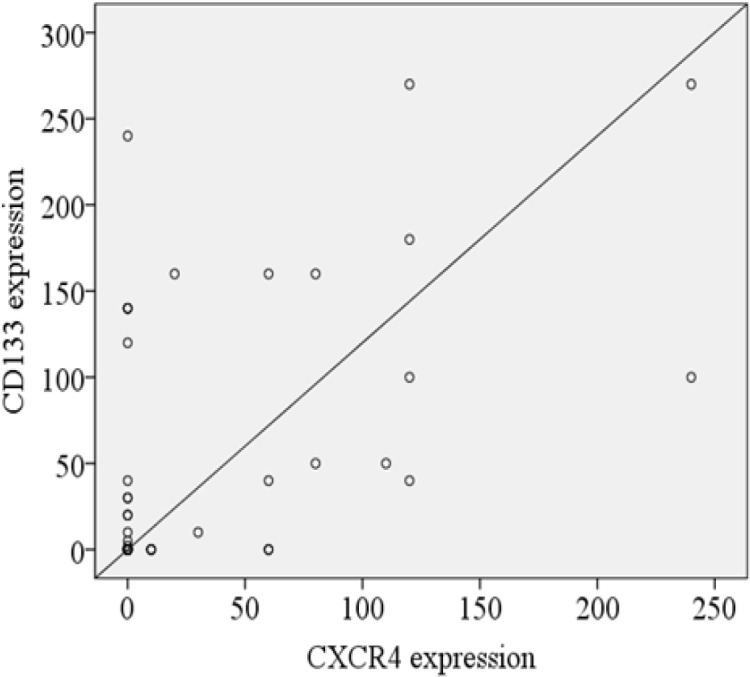
Box-Plot Graphics of CD133 and CXCR4 Expression Scoring (A) in Terms of Tumor Size, Necrosis, Stage and Metastasis. A plot shows mean (horizontal line), the lower and upper lines of the box 75th and 25th and outliers (circle)

*CD133* expression was observed in the cytoplasm and membrane of tumor cells and representative areas of immunostained OS tissues are shown in Figure 1. The number of *CD133*-positive cells considerably varied between different tumor samples. The stained sections were defined as *CD133* positive if *CD133* expression was detected in more than 10% of the whole tumor area. As shown in Table 1, *CD133* expression was classified based on the mean of *CD133* H-score which was 50 into low and high expression. High expression was found in 13/50 (26%) of OS cases. Of the 12 patients who showed lung metastasis, 5 cases belong to the high *CD133* expression group, and 7 cases were in the low *CD133* expression group with marginal trend (P=0.06). No significant correlation was observed between *CD133* expression and other clinicopathological characteristics listed in Table 1 and Figure 2.

*Correlation between CXCR4 expression and clinicopathological characteristics*

The different expression levels of the *CXCR4* was observed and indicated in Figure 1. Positive *CXCR4* expression was mainly detected in the cell membrane and cytoplasm of OS cells. A small number of OS cases showed *CXCR4* expression (36%) compared to negative group (64%). The expression of *CXCR4* was grouped based on the mean of* CXCR4* H-score as a cut off (mean= 31). The expression of *CXCR4* is presented in both groups as high (mean ≤31) and low (mean >31) expression. As indicated in Table 1, overall *CXCR4* expression was not significantly associated with gender, age, tumor size, necrosis, stage and metastasis (P>0.05).

*Association between CD133/CXCR4 markers expression with clinicopathological features of OS patients*

As indicated in Table 2, overall *CD133* and *CXCR4* examination using Pearson’s chi-square showed a significant reciprocal correlation between the expression patterns of the *CD133* and *CXCR4* (P=0.004). *CD133/CXCR4 *markers expression were classified into 4 groups including *CD113*High/*CXCR4* Low, *CD113* Low/*CXCR4* High, low expression of both markers (*CD133*Low/*CXCR4*Low), and high expression group (*CD133*High/*CXCR4*High). The association between *CD133*/*CXCR4 *phenotypes and clinicopathological factors were evaluated by the one-way ANOVA and Tukey’s post hoc analysis (Table 2). A significant association was observed between *CD133/CXCR4* phenotypes and lung metastasis, indicating that *CD133/CXCR4* expression was positively and equally related to lung metastasis as analyzed by the Tukey’s post hoc test (P=0.05). However, there was no significant association between the expression of *CXCR4 *and *CD133* and any of the other clinicopathological features listed in Table 2 (P>0.05).

**Table 1:** Correlation between CD133 Expression and Clinicopathological Factors in the Osteosarcoma Patients

Patients and tumor characteristics	No. of cases (%)	Expression of CD133		*P-value*	Expression of CXCR4		*P-value*
		low	high		low	high	
Mean age (yrs) ± SD	19±8.9						
≤19	24 (48)	19 (38)	7 (14)	1	20 (40)	6 (12)	0.75
>19	26 (52)	18 (36)	6 (12)		17 (34)	7 (14)	
Gender							
Male	29 (58)	22 (44)	7 (14)	0.75	24 (48)	5 (10)	0.11
Female	21 (42)	15 (30)	6 (12)		13 (26)	8 (16)	
Tumor size							
≤8	22 (44)	17 (34)	5 (10)	0.75	16 (32)	6 (12)	1
>8	28 (56)	20 (40)	8 (16)		21 (42)	7 (14)	
Enneking stage							
II	17 (34)	30 (60)	8 (16)	0.25	29 (58)	9 (18)	0.7
III	33 (66)	7 (14)	5 (10)		8 (16)	4 (8)	
Metastasis							
Yes	12 (24)	7 (14)	5 (10)	0.06	8 (16)	4 (8)	0.7
No	38 (76)	30 (60)	8 (16)		29 (58)	9 (18)	
Necrosis (%)							
≤90	23 (46)	15 (30)	8 (16)	0.21	18 (36)	5 (10)	0.74
>90	27 (54)	22 (44)	5 (10)		19 (38)	8 (16)	

**Table 2:** Correlation between CD133/CXCR4 Phenotypes and Clinicopathological Factors in the Osteosarcoma Patients

Patients and tumor characteristics	No. of cases (%)	CD133/CXCR4 phenotypes				*P-value*
		CD133Low/ CXCR4Low	CD133High/CXCR4Low	CD133Low/CXCR4High	CD133High/CXCR4High	
Mean age (yrs) ± SD	19±8.9					
≤19	24 (48)	16 (32)	4 (8)	3 (6)	3 (6)	0.87
>19	26 (52)	15 (30)	2 (4)	3 (6)	4 (8)	
Gender						
Male	29 (58)	20 (40)	4 (8)	2 (4)	3 (6)	0.43
Female	21 (42)	11 (22)	2 (4)	4 (8)	4 (8)	
Tumor size						
≤8	22 (44)	14 (28)	2 (4)	3 (6)	3 (6)	0.94
>8	28 (56)	17 (34)	4 (8)	3 (6)	4 (8)	
Tumor stage						
II	17 (34)	26 (52)	3 (6)	29 (58)	9 (18)	0.31
III	33 (66)	5 (10)	3 (6)	2 (4)	2 (4)	
Lung Metastasis						
Yes	10 (20)	3 (6)	3 (6)	2 (4)	2 (4)	0.05
No	40 (80)	28 (56)	3 (6)	4 (8)	5 (10)	
Necrosis (%)						
≤90	23 (46)	14 (28)	4 (8)	1 (2)	4 (8)	0.34
>90	27 (54)	17 (34)	2 (4)	5 (10)	3 (6)	

## Discussion

Osteosarcoma is the most common type of primary malignant bone tumor usually occurs during childhood or adolescence (Xie et al., 2016). Regardless of the long-term survival (68%), OS shows poor prognosis with high local aggressiveness and tendency for recurrence and metastasis especially to the lungs (Xie et al., 2016).

 Therefore, it is essential to elucidate underlying causes and markers involved in OS progression and metastasis and to find new therapeutic targets and approaches. It has been reported that OS metastasis play an important role in promotion of migratory tumor cells and metastatic niche generation at distant organs. Several factors via multifaceted processes and different signaling pathways involve in cancer metastasis (Lu et al., 2015b). Many studies have been focused on Chemokines as the main recruiting factors and on chemokine receptors produced by metastatic niche which lead to tumor cell attraction and homing in distant sites (Lazennec et al., 2010; Balkwill et al., 2004; Kryczek et al., 2007). *CXCR4* is an important role in the trafficking of normal and malignant cells to distant organs including the lymph nodes, lungs, liver and bone (Micucci et al., 2015). *CXCR4* as a CSC marker involved in tumor progression or poor survival of OS has been suggested by some studies (Sand et al., 2015; Xie et al., 2016). It has been reported that the high expression of CSC marker *CXCR4* correlated with significant prognostic value and therapeutic importance of RCC patients (D’Alterio et al., 2010). It has been revealed that CSCs exist in primary OS and cell lines derived from human OS tumors (Tirino et al., 2008; Gibbs et al., 2005). He at al showed that mRNA of the stemness genes such as octamer-binding transcription factor4 (Oct-4) and NANOG, and the metastasis-related receptor *CXCR4* were highly expressed in *CD133+* OS cells (He et al., 2012). They also showed that *CD133* expression as an independent prognostic factor was associated with lung metastasis and poor prognosis of OS patients (He et al., 2012). The current study was performed to assess the tissue expression pattern of* CD133* as a type of CSC marker and *CXCR4* as a metastasis-related marker in OS tumors using immunostaining method. To this end, we first defined that if OS tumors could express *CD133* and *CXCR4*. In our patient data, we found that high expression of *CD133* was partially associated with lung metastasis as a worse prognostic factor of OS. In line with our findings, He et al reported that high expression of *CD133* in OS tissues was associated with the high lung metastasis risk of OS (He et al., 2012). Based on previously published ﬁndings by Zambo et al., (2016) CD133 along with other CSC markers including nestin and *ABCG2* could be represented CSC phenotype in bone and soft tissue sarcomas. It has also been shown that high expression of *CXCR4* along with other stemness genes can affect the properties of *CD133+* cells invasion and migration supporting the proposed link between *CD133* expression and CSC characteristics (He et al., 2012). In a study performed by Lu et al the higher *CXCR4* and β catenin.

mRNA and protein expression rate was observed in OS cases than in the osteochondroma cases which were significantly correlated with the clinical Enneking stage, metastasis and survival of OS (Lu et al., 2015b). In other study of Lu et al significantly higher expression of *CXCR4* and β-catenin mRNA and protein was found in OS tissues compared to the adjacent non-neoplastic areas (Lu et al., 2015c). Another animal study showed that higher percentage of *CXCR4*-expressing lung metastatic cells compared to primary tumors is due to the important role of *CXCL12/CXCR4* axis in late metastasis (Neklyudova et al., 2016). Guo et al was revealed that coexpression of *CXCR4* with other factors such as HIF-1α through the hypoxia-HIF-1α-CXCR4 pathway has crucial function during the migration of human OS cells representing a novel targeting strategy specially for OS patients with distant metastasis (Guo et al., 2014). In Bai et al study using 22 patients with chondrosarcoma, higher *CXCR4* expression levels was observed in high-grade cells than in low-grade specimens (Bai et al., 2011). They suggested that their findings needed to be expanded and to be confirmed by a larger number of cases (Bai et al., 2011). High expression of *CXCR4* was revealed in canine OS cells with its signaling role in cell migration (Fan et al., 2008). To date, many studies have focused on the detection and possible prognostic significance of *CXCR4* as a reliable CSC marker in other cancers (Retz et al., 2005; Zhang et al., 2012). However, relatively little is known about the prognostic significance of *CXCR4* individually or in combination with other CSC markers in human OS. As previously revealed data were achieved using animal model, sarcoma cell lines and molecular evaluation (Walter et al., 2014; Zhang et al., 2013; de Nigris et al., 2008; Fan et al., 2008), our study has been more focused on the protein expression levels of two CSC markers *CD133* and *CXCR4* using immunohistochemical detection in the human OS tissues. Furthermore, we examined the possible relationship of *CD133* and *CXCR4* expression levels with metastasis as the main patients’ characteristic. Our immunohistochemical analysis of Iranian OS patients, for the first time, showed that despite the expression of *CXCR4* in tissues, no significant relationship between its expression and other clinical factors was observed. This discrepancy may be the result of variations in clones of antibodies, cut-off point and population study.

Co-expression of nestin, *CD133* and *ABCG2* as putative CSC markers has been studied in different type of sarcomas (Zambo et al., 2016). Co-expression of both CSCs markers of *CD133* and *CXCR4* has been presented in a variety of cancers, including colon cancer as markers for metastatic potential and poor prognosis (Silinsky et al., 2013; Li et al., 2015; Margolin et al., 2015), esophageal squamous cell carcinoma as a novel marker for predicting the poor prognosis of patients (Lu et al., 2015a), non-small cell lung cancer (NSCLC) as a prognostic marker via *CD133/CXCR4/EMT* axis (Tu et al., 2017), pancreatic adenocarcinoma (PA) (Mizukami et al., 2014), anaplastic thyroid carcinoma (ATC) (Yun et al., 2014) and renal cancer (RCC) (D’Alterio et al., 2010). Our study represented the first combined analysis of two putative CSCs markers expression together in human OS tissues and displayed their promising predictive values in OS. We found that *CD34High/CD117 *High phenotype was more frequent in OS cases.

Among 50 patient tissues, concomitant high *CD133-CXCR4* expression accounts for 20.78% (32/154). We also observed a positive significant correlation between *CD133/CXCR4* phenotype with OS lung metastasis. No significant correlation was found between co-expression of *CD133/CXCR4* with other clinicopathologic variables. The concomitant high *CD133-CXCR4* expression might be a novel marker for predicting the poor prognosis of patients with OS, and *CD133* and *CXCR4* may serve as potential therapeutic targets. Our preliminary data would argue for concomitant *CD133/CXCR4* immunostaining as a potential marker for biologic aggressiveness in estosarcoma of bone.

In conclusion, based on the here reported results regarding the synergistic effect of *CD133* and *CXCR4 *on metastasis and progression of OS, these two markers might be considered as potential therapeutic targets and favored over anti-metastatic systemic therapy.
